# Integration of the DIAMONDS and CAPTION situation taxonomies based on the South Korean culture

**DOI:** 10.3389/fpsyg.2024.1342133

**Published:** 2024-07-01

**Authors:** Heejeong Moon, Hyunnie Ahn

**Affiliations:** Department of Psychology, Ewha Womans University, Seoul, Republic of Korea

**Keywords:** situational taxonomy, wave 2 taxonomies, integrated situational taxonomy, DIAMONDS model, CAPTION model, bass-ackwards analysis

## Abstract

**Introduction:**

In this study, we developed an integrated situational taxonomy by merging the second-generation situational taxonomies of the DIAMONDS and CAPTION models.

**Method:**

The study participants included 363 adults aged 25–39 years, residing in South Korea, with at least a college degree, and currently employed. To integrate the factors of both models, we conducted an exploratory factor analysis and further examined the hierarchical structure of these factors using bass-ackwards analysis.

**Results:**

Our analysis revealed that the integrated situational taxonomy comprises seven domains with a confirmed hierarchical structure. Building on these findings, we further conducted a comparative analysis of the results with prior situational taxonomy research.

**Discussion:**

It was found that the factors constituting integrated domains derived from previous studies that employed correlation analysis or factor analysis differed from those of our study. However, the taxonomy of the three domains in the third level (negativity, positivity, and tasks) aligned with that of previous relevant research, suggesting that these domains are universally applicable to situational taxonomy. Hence, although the taxonomy of the three domains does not encapsulate specific situational characteristics, like the seven domains, if one seeks a culturally universal, statistically clear, and concise structure of situational taxonomy, the three-domain one is a promising alternative to the seven domains. Moreover, this study is the first situational taxonomic research outside the United States and European cultural spheres that confirms that an integrated situational taxonomy is similarly applicable in East Asian cultures.

## 1 Introduction

Scholars widely acknowledge that the two main factors influencing human behavior are *person* and *situation*. The influence of personal and situational factors on specific human actions has been termed as *person–situation debate* (Funder, 2009). Many studies in psychology have examined the core components of personality, which are key factors influencing human behavior. Personality trait theorists assert that personality comprises five factors: *agreeableness, conscientiousness*, *extraversion*, *neuroticism*, and *openness* (Allport, 1937; Guilford, 1975) However, research aimed at identifying the fundamental nature of situations and their constituent elements has progressed slower than personality research (Rauthmann et al., 2015). Scholars have explored how various situations affecting human behavior might be organized; following Rauthmann et al. (2014), they have investigated situational taxonomies to classify situations with similar properties, with seven emerging situational taxonomies: DIAMONDS (Rauthmann et al., 2014), CAPTION (Parrigon et al., 2017), Situational Affordances for Adaptive Problems (SAAP; Brown et al., 2015), Situation 5 (Ziegler et al., 2019), Social Interdependence Scale (SIS; Gerpott et al., 2018), Situation Six (Oreg et al., 2020), and the Big5 Framework (Griffo and Colvin, 2019). These taxonomies are known as *Wave 2 taxonomies*. Although certain situational dimensions are consistent across most taxonomies, scholars have identified dimensions unique to Wave 2 taxonomies (Rauthmann et al., 2020). This underscores the need to develop an integrated taxonomy of situations based on similarities among the dimensions that constitute diverse situational taxonomies. In this study, we used an integrative approach to the DIAMONDS (duty, intellect, adversity, mating, pOsitivity, negativity, deception, and sociality) and CAPTION (complexity, adversity, positive valence, typicality, importance, humOr, and negative valence) models among the seven Wave 2 situational taxonomies to derive a unified situational taxonomy model, using a domestic Korean sample to ensure validity. The integration of DIAMONDS and CAPTION models among the seven Wave 2 taxonomies was prioritized because they have more diverse prior research on integration than other models (Rauthmann and Sherman, 2018, 2019). Moreover, they have been validated with Korean samples (Moon, 2022). Previous situational taxonomic research has predominantly focused on Western cultures, whereas research across diverse cultures is limited. This study was the first situational taxonomic research outside the United States and European cultural spheres. Consequently, we aimed to conduct a technical-level descriptive comparison between the results of the integrated situational taxonomy developed in this study and those of previous similar research conducted in Western cultures.

### 1.1 Theoretical background

#### 1.1.1 Definition and types of situations

Despite the fact that personality and situation are two major factors influencing human behavior, scholars have yet to reach a clear consensus on the definition of “situation” (Hogan, 2009). This lack of agreement is due to the complexity inherent in any given situation, which contains various types of information and features.

Traditionally, situations can be divided into objective and subjective ones. The objective aspect of a situation is based on physically existing conditions, that is, on objective information that constitutes a situation (e.g., when, where, who, what, how). From this perspective, an objective situation can be defined as the “comprehensive conditions coexisting at a given place and time” (Pervin, 1978). This aspect of a situation possesses objectivity in that it yields the same evaluation regardless of who assesses the situation (Maclver, 1942).

On the other hand, subjective situations, also referred to as psychological situations, focus on an individual’s unique experiences and perceptions of what thoughts and emotions they experience within a situation (Rauthmann et al., 2015). In essence, a subjective situation expresses the subjective characteristics of a situation based on the meanings and interpretations that an individual ascribes to an objective situation. Therefore, even in the same situation, its evaluation can differ depending on the individual’s perception. For instance, one person may perceive a business meeting as exhausting and stressful, whereas another may see it as productive and challenging. In this way, subjective situations reflect the individual’s unique psychological characteristics.

#### 1.1.2 Early research on situational classification

A primary goal of psychological research is to understand and predict a wide range of human behaviors. Consequently, comprehending the fundamental characteristics of situations that influence human behavior remains a crucial task in psychology. For this purpose, research into a basic and concise taxonomic structure of the core components of situations is necessary. Once such a classification system is developed, it will not only allow for the systematic differentiation of various phenomena by describing the key attributes of situations but also provide a detailed understanding of the role situations play in manifesting personality into behavior (Reis, 2008).

Based on this necessity, efforts to elucidate and classify the attributes of situations began in the 1960s. However, at that time, due to the lack of clear consensus on the components of situations, research primarily focused on relatively specific situations (e.g., leisure activities, religious practices) or situations experienced by particular groups (e.g., situations primarily encountered by housewives) and their impact on human behavior (Yang et al., 2009). Moreover, early research on the classification of situations often failed to present empirical results (Rauthmann and Sherman, 2020), largely because tools for measuring situation classification had not yet been developed. In other words, the absence of measurement tools for situations made it difficult to repeatedly measure derived situational classifications within a single study, thus rendering the comparison and analysis of these results with subsequent research findings impossible (Rauthmann et al., 2020).

#### 1.1.3 Wave 2 taxonomies

Recent efforts to overcome the limitations of early situational classification methodologies have led to significant advancements following the development of a measurement tool for classifying situations by Rauthmann et al. (2014). Subsequent classifications developed after this research are referred to as the “Wave 2 taxonomy.” The seven models and their constituent factors included in Wave 2 taxonomies are presented in Table 1. Wave 2 taxonomies possess several characteristics that distinguish them from earlier situational classifications.

**Table 1:** Wave 2 taxonomies.

**Taxonomy**	**Reference**	**Number of factors**	**Factors**	**Tradition**	**Measurement tool**
DIAMONDS	Rauthmann et al. (2014)	8	Duty, Intellect, Adversity, Mating, pOsitivity, Negativity, Deception, Sociality	Riverside Situational Q-sort	RSQ-8
CAPTION	Parrigon et al. (2017)	7	Complexity, Adversity. Positive valence, Typicality, Importance, HumOr, Negative valence	Lexical (English)	CAPTIONs, CAPTIONs-SF
SAAP	Brown et al. (2015)	7	Self-protection, Disease Avoidance, Affiliation, Status, Mate seeking, Mate retention, Kin care	Evolutionary Theory	SAAP
Situation 5	Ziegler et al. (2019)	5	Outcome-expectancy, Briskness, Psychological + physical load, Lack of stimuli, Cognitive load	Lexical (German)	B5PS
SIS	Gerpott et al. (2018)	5	Interdependence, Conflict, Power, Future interdependence, Information certainty	Interdependence Theory	SIS
Situation Six	Oreg et al. (2020)	6	Negativity, Positivity, Familiarity, Demandingness, Oddness, Straightforwardness	Lexical (Hebrew)	Situation Six Questionnaire
Big5 Framework	Griffo and Colvin (2019)	5	Dominance/aggression, Negative affect, Achievement, Positive affect/affiliation, Situation strength	Trait Theory	Big5 Framework Questionnaire

The primary characteristic of Wave 2 taxonomies is their focus on subjective rather than objective situations (Rauthmann et al., 2014; Parrigon et al., 2017). A subjective situation is constructed from an individual’s personal interpretation of the objective information that constitutes a situation. The subjective information deemed meaningful by an individual in a specific context is referred to as “situation characteristics.” Situation characteristics refer to the subjective information that individuals consider significant in a particular situation. As situation characteristics are derived from individuals’ subjective evaluations of objective situations, psychologists increasingly evaluate them as essential elements for understanding human behavior (Mischel and Shoda, 1995; Edwards and Templeton, 2005).

Early situational classification research has primarily aimed at classifying components of situations. Conversely, recent research on situational taxonomy has introduced taxonomies directly relevant to understanding human behavior. Recent situational research has sought to use situational classification to enhance the understanding of personality-related behaviors (Rauthmann et al., 2014; Griffo and Colvin, 2019; Oreg et al., 2020), dynamics of everyday behavior (Rauthmann and Sherman, 2015), evolutionary issues (Brown et al., 2015), and social interactions (Gerpott et al., 2018).

Concise and practical tools have been developed to measure situational taxonomies. Early situational classification research has primarily presented conceptual classifications of situations, with limited empirical research on situational classification (Rauthmann and Sherman, 2020). The main reason empirical research has been lacking is the absence of tools to measure these classifications. With the development of scales to measure Wave 2 situational taxonomies, research has become more diversified in terms of the types of situations, study participants, and research methods compared to early studies.

#### 1.1.4 Integration of situational taxonomies

The emergence of various Wave 2 situational taxonomies enriched our understanding of the systems and structures of these situations. However, an integrated situational taxonomy that considers the features of all individual taxonomies and possesses both reliability and validity is still needed because of the differences in the dimensions. In particular, factor analyses targeting all factors comprising the various situational taxonomies developed to date are required to develop an integrated situational taxonomy with reliability and validity. Nevertheless, only factor analysis studies integrating the components of the DIAMONDS and CAPTION models have been conducted (Rauthmann and Sherman, 2019), and factor analyses on integrating other classification methods have not yet been attempted.

Various attempts have been made to classify factors with similar characteristics by examining the correlations among factors comprising multiple situation taxonomies. Specifically, during the development of Wave 2 taxonomies, efforts were made to confirm the relationship between the components of the DIAMONDS model and factors of the newly developed Wave 2 taxonomies (Parrigon et al., 2017; Ziegler et al., 2019; Oreg et al., 2020). Based on the relationships and conceptual similarities among factors derived from these previous studies, Rauthmann and Horstmann (2019) attempted to classify the 43 factors comprising DIAMONDS, CAPTION, SAAP, Situation 5, SIS, Situation Six, and the Big5 Framework into six domains, termed Replicable 6: situations involving external threats (Treat), situations requiring responses to negative events (Stress), situations related to work (Tasks), situations requiring cognitive information processing (Processing), enjoyable situations (Fun), and mundane situations related to everyday life (Mundane).

The significance of the study conducted by Rauthmann and Horstmann (2019) lies in its consideration of the relationships among all factors comprising the seven Wave 2 situational taxonomies developed to date, reflecting the ultimate goal of situational researchers to integrate these taxonomies. However, the method of classifying factors based solely on conceptual associations and correlations adopted by Rauthmann and Horstmann (2019) has limitations in securing statistical reliability and validity. Therefore, many scholars have suggested conducting integration studies among models using factor analysis methods (Rauthmann et al., 2020). Nevertheless, integrating the 43 factors comprising the seven models through a single factor analysis study may be impractical, as issues such as sample collection and research design complexity may arise. Hence, scholars propose a gradual integration of various models starting with the integration of two or three models (Rauthmann and Sherman, 2018; Rauthmann et al., 2020). This study aimed to examine the integration of the DIAMONDS and CAPTION models, which have already been integrated through factor analysis methods in the study by Rauthmann and Sherman (2019), targeting Korean samples and building upon the validity established by Moon (2022).

#### 1.1.5 The DIAMONDS model

The DIAMONDS model was developed through a factor analysis of the Riverside Situational Q-sort (RSQ), which presents 89 broad and comprehensive situations that can be experienced in everyday life (Wagerman and Funder, 2009; Sherman et al., 2010; Funder, 2016). The RSQ was the first validated tool designed to measure the psychological characteristics of situations, and it assesses a wide array of situational content. Moreover, its items are associated with personality traits encompassing all the Big Five personality traits (Sherman et al., 2010). Factor analysis of the 89 situations presented in the RSQ resulted in eight situational dimensions (the RSQ-8) labeled *duty*, *intellect*, *adversity*, *mating*, *positivit*y, *negativity*, *deception*, and *sociality* (Rauthmann et al., 2014).

*Duty* pertains to situations in which one must fulfill a given role or task, solve problems, or make decisions. *Intellect* refers to situations requiring intellectual engagement, deep introspection, or cognitive capabilities. *Adversity* refers to situations in which one faces specific threats, encompassing conflicts, competitions, criticisms, blame, and sacrifices. *Mating* involves situations related to romantic or potential sexual partners, and the degree to which such situations are perceived as being conducive to romantic and sexual relations. *Positivity*, a dimension commonly derived in situational taxonomy research, assesses how potentially pleasant, enjoyable, or straightforward a situation is. *Negativity*, by contrast, describes the potential of a situation to induce negative emotions such as frustration, anxiety, and anger. It is a dimension, consistently found in situational taxonomy studies. *Deception* concerns situations in which individuals might be aware of mistrust, hostility, or lies that could damage mutual trust. Finally, *sociality* signifies situations involving communication and interaction with others, where one can experience warmth or relief by forming relationships.

#### 1.1.6 The CAPTION model

The CAPTION model is a situational taxonomy derived from the factor analysis of approximately 50 million adjectives that can describe the characteristics of a situation. These adjectives were extracted from subtitles from an American movie made between 1900 and 2007 (Parrigon et al., 2017). Although the CAPTION model was developed based on a lexical approach, it shares similarities with the DIAMONDS model in terms of the use of words that comprehensively describe situational features. In the CAPTION model, situations are categorized into seven dimensions called complexity, adversity, positive valence, typicality, importance, humor, and negative valence.

Complexity encompasses situations perceived as being emotionally or ethically intricate and those demanding cognitive skills, such as learning or deep thinking. Adversity relates to situations that deplete physical or psychological resources, thus making problem-solving challenging and inducing stress and burden. Positive valence is associated with situations that are perceived positively, including daily occurrences that elicit positive emotions or instances in which one experiences intimacy in interpersonal relationships. Typicality refers to commonplace situations that, because of their familiarity, are not perceived as novel or intriguing. Importance denotes situations that are generally significant or perceived as crucial for achieving individual goals, typically involving goal-oriented behaviors such as effort or focus. Humor concerns the degree to which a situation is perceived as humorous or lighthearted, encompassing both the positive aspect of being fun and playful and the negative potential of offending someone. Negative valence describes situations that are potentially harmful or threatening. It bears similarities with factors in second-generation situational classification research that signify stress-induced threatening situations.

#### 1.1.7 Association between the DIAMONDS and CAPTION models

Rauthmann and Sherman (2018) considered conceptual similarities between the dimensions of the DIAMONDS and CAPTION models to integrate highly correlated dimensions. This integration resulted in the identification of five domains: Domain 1 (*threats*) relates to situations involving social threats or conflicts; Domain 2 (*stress*) pertains to situations that evoke negative emotions; Domain 3 (*tasks*) involves situations demanding tasks or work completion; Domain 4 (*processing*) requires analytical and cognitive reasoning; and Domain 5 (*fun*) is associated with situations eliciting positive emotions. Notably, the sociality and mating dimensions of the DIAMONDS model and the typicality dimension of the CAPTION model were not distinctly incorporated into these five domains. Rauthmann and Sherman (2019) integrated the DIAMONDS and CAPTION models using factor analysis, thereby leading to the extraction of seven domains. Although the factor analysis results differed somewhat from their prior correlation analysis (Rauthmann and Sherman, 2018), domains resembling domains 1–5 were identified. Additionally, sociality and typicality, which showed no clear correlation in the correlation study, were classified as distinct dimensions in the factor analysis. In our analysis, Domain 6 was labeled *sociality,* and Domain 7, *typicality*.

#### 1.1.8 Hierarchical structure of the integrated situational taxonomy

Merging various taxonomies that measure a single concept requires considering hierarchical relationships among factors (Wright and Simms, 2014). The model structure reflecting inter-factor relationships can be broadly categorized into *list* and *hierarchical* organizations (Loehlin and Goldberg, 2014). Usually, a list organization represents a parallel arrangement of independent factors constituting a specific concept with no further subdivision (e.g., The Big Five traits: Agreeableness, Conscientiousness, Extraversion, Neuroticism, and Openness). Conversely, hierarchical organizations can divide abstract higher-order factors into more concrete lower-order factors. Discrepancies among model factors regarding a specific concept can arise because of differing levels of generalization (Guilford, 1975). For instance, dimensions such as the Situation Affordances for Adaptive Problems (SAAP) model’s affiliation, status, mate-seeking, mate-retention, and kin care dimensions; the DIAMONDS model’s mating and sociality dimensions; and the Situational Interdependence Scale’s (SIS’s) interdependence dimension all pertain to situations related to positive interpersonal relationships. However, the SAAP dimensions measure more specific situations than the DIAMONDS and SIS models, thus leading to discrepancies. When conducting hierarchical analyses of models with differing levels of generalization, we can expect more detailed situational dimensions to emerge as the analysis progresses. Conducting hierarchical analyses of the dimensions from various situational taxonomies can clarify the structure of integrated situational taxonomies and enhance our understanding of the differences between taxonomies (Wright and Simms, 2014).

#### 1.1.9 Need for cross-cultural comparative research on integrated situational taxonomy

This study aimed to integrate the existing factors of the DIAMONDS and CAPTION models to derive a new integrated situational taxonomy, particularly focusing on identifying the hierarchical structure among the constituent factors. Previous attempts have been made to integrate the factors of the DIAMONDS and CAPTION models (Rauthmann and Sherman, 2018, 2019). However, as these studies targeted Western samples, the integrated situational taxonomy in non-Western cultural spheres is unknown.

Both the DIAMONDS and CAPTION models classify situation characteristics, reflecting individuals’ subjective perceptions of situations. Therefore, cultural validity is important considering the validity of Wave 2 taxonomies (Kikutani et al., 2016). Cultural validity refers to the suitability and usefulness of a theory developed in a specific cultural context when applied to research subjects from different cultural backgrounds (Leong and Chou, 1997). As situation characteristics reflect individuals’ subjective perceptions of situations, interpretations of situations may vary across cultures due to differences in individuals’ cultural backgrounds (Nisbett and Norenzayan, 2002).

Prior research has already demonstrated that the interaction between situations and culture significantly impacts human behavior. Specifically, depending on their cultural backgrounds, individuals may perceive the same situation differently (Rauthmann et al., 2014). For example, in studies involving participants from the United States, the United Kingdom, Japan, and Cambodia, only the Americans reported experiencing both positive and negative emotions in situations typically associated with fear (Kikutani et al., 2016). This can be interpreted as reflecting Americans’ preference for high-arousal sensation-seeking activities (Tsai, 2007).

Considering the importance of cultural validity, this study aimed to identify differences between the results of integrated situational taxonomy research targeting Korean individuals and that targeting Western individuals. Furthermore, we explored the universality and cultural specificity of the components of situational taxonomies according to the cultural context.

Based on the necessity and theoretical foundation outlined, this study aimed to examine whether the integrated situational taxonomy of the DIAMONDS and CAPTION models, previously applied to Western samples, can also be applicable within the Korean cultural context. To this end, the current study initially integrated the factors comprising the DIAMONDS and CAPTION models targeting a Korean sample, in accordance with prior research (Rauthmann and Sherman, 2019), to develop a unified integrated situational taxonomy. Additionally, to more specifically ascertain the derivation process of the final dimensions constituting this taxonomy, this study intended to analyze the hierarchical structure among these dimensions using the bass-ackwards method. Based on these statistical analyses, a comparative discussion of the integrated situational taxonomy derived from previous research and the findings of this study was conducted in the discussion and conclusions section. The specific research questions were as follows:

RQ*_1_*: What domains does the integrated situational taxonomy with factors comprising the DIAMONDS and CAPTION models targeting Korean individuals consist of?

RQ*_2_:* What hierarchical structure do the integrated dimensions of the DIAMONDS and CAPTION models targeting Korean individuals exhibit?

RQ*_3_:* What are the similarities and differences between the findings of the integrated situational taxonomy study conducted on Western samples and those of the current study conducted on Korean samples?

## 2 Methods

### 2.1 Participants

To integrate the DIAMONDS and CAPTION models, data were collected through an online survey agency. The study participants provided written informed consent for their participation. The survey took approximately 15 min to complete, and participants were offered a token of appreciation worth about $2 upon survey completion. Considering previous research findings that individuals’ experiences of situations vary depending on demographic characteristics, such as age and education (Brown and Rauthmann, 2016), this study restricted the sample to adults aged 25–39 living in South Korea, who were employed and had at least a college degree, to minimize the influence of demographic variables on the research outcomes. Furthermore, considering that it is desirable to have a sample size of at least five times the number of items when conducting factor analysis (Tabachnick et al., 2007), the aim for this study was to collect data from over 300 participants, based on a total of 60 items from the RSQ-8 and the Korean adaptation of the CAPTIONs-SF, a short form of the CAPTION scale. Finally, data were collected from 363 participants (*M*_age_ = 32.8, *SD =* 4.1, 53.7% women).

### 2.2 Measures

#### 2.2.1 Riverside situational Q-sort-8

To measure the DIAMONDS model, the Riverside situational Q-sort-8 (RSQ-8)—which reflects the characteristics of its eight dimensions (Rauthmann et al., 2014)—was adapted into Korean. This scale comprises 32 items measuring the dimensions of duty, intellect, adversity, mating, positivity, negativity, deception, and sociality. Each item on the RSQ-8 was evaluated using a seven-point Likert scale. In this study, the internal consistency reliability values for the DIAMONDS model factors were as follows: duty, 0.89; intellect, 0.85; adversity, 0.92; mating, 0.84; positivity, 0.93; negativity, 0.94; deception, 0.92; and sociality, 0.89. Moon (2022) validated the RSQ-8 with the same sample as this study using a confirmatory factor analysis (CFA). The results indicated good fit indices: χ2 = 910.40 (*df* = 436, *p* < 0.001), CFI = 0.92, TLI = 0.91, SRMR = 0.06, and RMSEA = 0.06, all meeting the criteria for good fit.

#### 2.2.2 CAPTION scale short form (CAPTIONs-SF)

To measure the CAPTION model for this study, the Korean adaptation of the CAPTIONs-SF, a short form of the CAPTION scale developed by Parrigon et al. (2017) using Hebrew adjectives, was employed. It has 28 items measuring seven factors, complexity, adversity, positive valence, typicality, importance, humor, and negative valence, and uses a seven-point Likert scale for the responses. The internal consistency reliability values for the CAPTION model factors were as follows: complexity, 0.93; adversity, 0.91; positive valence, 0.93; typicality, 0.86; importance, 0.93; humor, 0.92; and negative valence, 0.96. Moon (2022) assessed the validity of CAPTIONs-SF using the same sample as this study using a CFA. The results revealed approximate fit indices: χ2 = 1200.30 (*df* = 329, p < 0.001), CFI = 0.89, TLI = 0.88, SRMR = 0.09, and RMSEA = 0.09, all indicating satisfactory fit.

### 2.3 Procedures

This study was approved by the institutional review board of Ewha Womans University (Approval No: [ewha-202110-0008-01]). The original English RSQ-8 and CAPTIONs-SF scales were translated into Korean. One individual with a PhD in psychology and one doctoral candidate in psychology translated the scales into Korean, which were then back-translated into English by a psychology master’s student. Finally, the same PhD and doctoral students evaluated the equivalence between the original and back-translated versions, thus completing the scale adaptation. To investigate the integration of the DIAMONDS and CAPTION models, participants first answered demographic variables and then assessed specific situations using the RSQ-8 and CAPTIONs-SF items. To select specific situations for evaluation, participants were randomly presented with one of three time slots: yesterday at 11AM, 3PM, or 7PM. They then recalled a specific situation that occurred at a designated time and then responded to open-ended questions about the location where the situation occurred, the people present, and their actions during that situation. Subsequently, the participants evaluated how well the multiple-choice items of the RSQ-8 and CAPTIONs-SF reflected the characteristics of the recalled situation described in their open-ended responses.

### 2.4 Analyses

This study aimed to review the structural relationships of the factors comprising the DIAMONDS and CAPTION models and ultimately derive an integrated situational taxonomy. Individual dimensions of the DIAMONDS and CAPTION models are referred to as *factors*, whereas new integrated factors derived from combining the two models are termed as *domains*.

After the survey, descriptive statistical analysis was conducted to confirm the demographic characteristics of the participants, followed by descriptive statistics and correlation analyses of the RSQ-8 and CAPTIONs-SF results. The Cronbach’s α coefficient by factor was used to confirm the internal consistency reliability of each scale. The analyses were performed using the IBM® SPSS® Statistics 25 statistical software package.

Next, an exploratory factor analysis (EFA) was conducted on the average scores of the items constituting each of the 15 individual factors of the DIAMONDS and CAPTION models to confirm the parallel structure based on conceptual commonality. This helped us understand the direction of subsequent hierarchical analysis research. Based on previous research, principal axis factoring was used to minimize data loss, and owing to the anticipated significant correlations between factors (Rauthmann and Sherman, 2019), the direct oblimin method was employed. The optimal number of factors extracted from the results was determined by considering interpretability and statistical guidelines (eigenvalues, scree plots, and cumulative variance ratios; Wright et al., 2012). IBM SPSS Statistics 25 was used for all EFAs.

The hierarchical structure of the integrated situational taxonomy was then assessed using a top–down approach employing the bass-ackwards method (also known as sequential factor analysis) to extract the principal components sequentially (Goldberg, 2006). This method is the only way to understand the multiple hierarchical levels of domains derived from EFA (Michelini et al., 2019). The bass-ackwards method starts with a super factor encompassing all components in the first hierarchy and separates lower-order factors from higher-order ones through a principal component analysis at each step (Tackett et al., 2008). Typically, when the path coefficient, a correlation between factor scores of adjacent hierarchies, is above 0.90, the factor is tentatively considered a single factor that no longer divides (Tackett et al., 2008). The bass-ackwards method for the hierarchical analysis was performed using the IBM SPSS Statistics 25 and Mplus 7 software packages.

## 3 Results

### 3.1 Descriptive analysis

To integrate the DIAMONDS and CAPTION models, we examined the mean, standard deviation (*SD*), skewness, and kurtosis for the scales of each model. The mean ranged between 1.94 and 4.45 and *SD* distributed between 1.20 and 1.62. The absolute values for skewness ranged from 0.07 to 1.4 and kurtosis values ranged from 0.23 to 2.57. Data are considered to meet the normality assumption when the absolute value of skewness is <2 and the absolute value of kurtosis is <7 (Curran et al., 1996). The analysis results indicated that all factors of the DIAMONDS and CAPTION models met the normality assumption.

Thereafter, we conducted a correlation analysis between the factors of the DIAMONDS and CAPTION models (Table 2). The correlation coefficients among the DIAMONDS model’s factors ranged from −0.22 to 0.72, whereas those of the CAPTION model ranged from −0.31 to 0.62. Correlation coefficients between the factors of the DIAMONDS and CAPTION models ranged from −0.49 to 0.69. Notably, the factors that exhibited strong correlations (greater than 0.60) included the relationship between the domains of negativity from the DIAMONDS model and adversity from the CAPTION model; positivity from the DIAMONDS model and positive valence from the CAPTION model; and adversity, negativity, and deception from the DIAMONDS model and negative valence from the CAPTION model.

**Table 2:** Correlation between factors of the DIAMONDS and CAPTION models.

	**1**	**2**	**3**	**4**	**5**	**6**	**7**	**8**	**9**	**10**	**11**	**12**	**13**	**14**	**15**
1. Duty (D)	1														
2. Intellect (D)	0.67^***^	1													
3. Adversity (D)	0.28^***^	0.46^***^	1												
4. Mating (D)	0.14^**^	0.38^***^	0.72^***^	1											
5. Positivity (D)	−0.16^**^	0.05	−0.01	0.25^***^	1										
6. Negativity (D)	0.37^***^	0.42^***^	0.66^***^	0.49^***^	−0.22^***^	1									
7. Deception (D)	0.26^***^	0.36^***^	0.66^***^	0.56^***^	−0.01	0.69^***^	1								
8. Sociality (D)	0.20^***^	0.31^***^	0.18^***^	0.34^***^	0.39^***^	0.11^*^	0.29^***^	1							
9. Complexity (C)	0.53^***^	0.59^***^	0.38^***^	0.33^***^	−0.04	0.47^***^	0.39^***^	0.23^***^	1						
10. Adversity (C)	0.48^***^	0.34^***^	0.50^***^	0.24^***^	−0.49^***^	0.69^***^	0.55^***^	0.02	0.48^***^	1					
11. Positive valence (C)	−0.12^*^	0.09^*^	0.01	0.27^***^	0.66^***^	−0.14^**^	−0.04	0.47^***^	0.09	−0.31^***^	1				
12. Typicality (C)	0.14^**^	0.07	−0.06	0.05	0.15^**^	−0.07	−0.02	0.11^*^	0.17^**^	0.04	0.24^***^	1			
13. Importance (C)	0.28^***^	0.31^***^	0.02	0.16^**^	0.35^***^	0.01	0.04	0.28^***^	0.44^***^	−0.01	0.47^***^	0.44^***^	1		
14. Humor (C)	−0.05	0.22^***^	0.36^***^	0.45^***^	0.40^***^	0.21^***^	0.29^***^	0.29^***^	0.22^***^	0.02	0.46^***^	0.06	0.20^***^	1	
15. Negative valence (C)	0.22^***^	0.32^***^	0.66^***^	0.45^***^	−0.21^***^	0.63^***^	0.60^***^	0.10^*^	0.44^***^	0.62^***^	−0.02	−0.03	−0.01	0.43^***^	1

(D), DIAMONDS model; (C), CAPTION model; *p < 0.05, **p < 0.01, ***p < 0.001.

### 3.2 Exploratory factor analysis

To examine what domains constitute the newly formed integrated situational taxonomy, we performed an EFA on the 15 factors included in the DIAMONDS and CAPTION models, using a Korean sample (RQ1). The EFA was conducted to integrate the factors comprising both models and derive situational domains that are statistically reliable, valid, and psychologically meaningful. In particular, this study aimed to integrate factors between the two models rather than integrating items within each model. Therefore, item parcels for the 15 factors were created, and EFA was conducted to maintain each factor of the two models. Item parceling is a method commonly employed in structural equation modeling to enhance model fit and estimation accuracy by aggregating measurement variables into one factor, typically by averaging or summing multiple items. In this study, mean rather than total scores of frequently endorsed items were used as indicator variables (Little et al., 2013).

First, we verified the suitability of the data for EFA by checking the Kaiser–Meyer–Olkin (KMO) index and conducting Bartlett’s test of sphericity. Generally, a KMO index above 0.90 is considered excellent, a value above 0.80 is good, and a value below 0.50 is deemed unsuitable for factor analysis (Cerny and Kaiser, 1977). The KMO value for our data was 0.86, indicating adequacy. Bartlett’s test of sphericity produced an χ^2^ value of 3128.65 (*p* < 0.001), thus confirming the data’s suitability for factor analysis.

Thereafter, we conducted EFA on the 15 factors. The principal axis factoring method was adopted for factor extraction, as it minimizes information loss while summarizing the content (Fabrigar et al., 1999). For the rotation, we employed the direct oblimin method, assuming correlations between the factors.

Generally, to determine the number of factors in EFA for new scale development, eigenvalues, scree plots, cumulative variance ratios, interpretability, and results from previous research are used (Kim, 2016). Because our study aimed to validate whether the factors derived from the DIAMONDS and CAPTION models through previous research could also be factored into a domestic Korean sample, we prioritized the results from previous research when deciding on the number of factors. Given that seven domains have been identified in previous research (Rauthmann and Sherman, 2019), our study also compared models with five to seven factors, considering interpretability, eigenvalues, scree plots, and cumulative variance ratios. Additionally, we also considered whether individual factors loaded more than 0.40 on one construct and less than 0.30 on others (McCoach et al., 2013).

The EFA results for the DIAMONDS and CAPTION models were verified using scree plots. The plot showed three factors with eigenvalues above one, which deviated from the seven factors identified in a previous study (Rauthmann and Sherman, 2019). Considering the scree plot results, we found three major domains when analyzing the three-factor model: generally negative situations (e.g., adversity from the DIAMONDS model, negative valence from the CAPTION model), generally positive situations (e.g., positive valence from the DIAMONDS model, positivity from the CAPTION model), and work-related situations (e.g., duty from the DIAMONDS model, complexity from the CAPTION model). However, several distinct factors are conceptually grouped into one domain, which makes it challenging to differentiate between specific situational characteristics and daily experiences. Additionally, the cumulative variance ratio did not reach the recommended value of 60%. Conversely, considering five to seven factors based on previous research, although individual factors had eigenvalues below one, the cumulative variance ratio exceeded 60% for models with five or more factors. In the models with five to seven factors, all factor loadings were above 0.40, thus establishing the validity of the model. On the basis of these statistical considerations and the interpretability of the models derived from previous research, we selected a model with seven factors (Table 3).

**Table 3:** Exploratory factor analysis results of the integrated situational taxonomy.

	**Factor**						
	**1**	**2**	**3**	**4**	**5**	**6**	**7**
(D) Negativity	0.827	0.173	−0.033	0.208	0.096	−0.048	−0.055
(C) Adversity	0.806	0.284	−0.275	0.024	−0.035	−0.050	0.021
(D) Deception	0.798	0.082	0.074	0.178	0.008	0.249	0.025
(C) Negative valence	0.782	0.131	0.229	0.240	−0.138	−0.061	−0.101
(D) Intellect	0.194	0.878	0.155	0.171	0.130	0.049	−0.021
(D) Duty	0.216	0.873	−0.178	0.000	0.081	0.090	0.121
(C) Humor	0.266	0.058	0.888	0.153	−0.038	0.056	0.016
(D) Positivity	−0.356	−0.118	0.639	0.112	0.355	0.265	0.018
(C) Positive valence	−0.227	−0.090	0.550	0.168	0.442	0.423	0.098
(D) Mating	0.286	0.072	0.201	0.853	0.108	0.174	0.022
(D) Adversity	0.571	0.199	0.116	0.673	−0.039	−0.044	−0.023
(C) Importance	−0.057	0.216	0.140	0.014	0.822	0.115	0.283
(C) Complexity	0.445	0.514	−0.029	0.101	0.533	0.027	−0.080
(D) Sociality	0.087	0.137	0.176	0.087	0.108	0.930	0.033
(C) Typicality	−0.057	0.056	0.035	0.005	0.188	0.038	0.963
Cronbach’s *α*	0.95	0.91	0.92	0.89	0.87	0.88	0.85
Eigenvalue	4.90	2.97	1.83	0.92	0.76	0.59	0.58
Explained variance	23.56	13.82	11.97	9.50	9.32	8.31	7.05
Cumulative rate	23.56	37.37	49.34	58.84	68.15	76.46	83.50

The results of the EFA indicated that Domain 1 includes the negativity and deception of DIAMONDS and adversity and negative valence of CAPTION. Domain 1 encompasses situations commonly encountered in daily life that lead to the depletion of physical and psychological resources and consequently induce negative emotions. Thus, Domain 1 was labeled *negativity*. Domain 2 comprises duty and intellect factors of DIAMONDS, encompassing situations wherein intellectual capabilities are applied to perform certain tasks; this domain was labeled as *tasks*. Domain 3 consists of the positivity factor of DIAMONDS and positive valence and humor factor of CAPTION. All these factors were associated with situations that evoke positive emotions such as humor, playfulness, joy, warmth, and value; hence, it was labeled *positivity*. Domain 4 incorporates the mating and adversity dimensions from the DIAMONDS model. Mating describes situations that might arise in romantic or sexual relationships with the opposite sex, while adversity pertains to feelings of being criticized or controlled. Given the high loadings for these two dimensions and their inter-correlation within Domain 4, this domain may relate to situations in which one feels criticized or controlled by a partner or spouse. Additionally, many items measuring the mating dimension pertain to sexual encounters, suggesting that Domain 4 could also encompass unwanted or risky sexual situations. Thus, Domain 4 was labeled as *threats*. Domain 5 includes the importance and complexity factors of CAPTION. Domain 5 seemed to relate to situations in which analytical and scholarly methods are used effectively and beneficially and was labeled *resolution*. Domain 6, consisting solely of the sociality factor from the DIAMONDS, describes social interactions. Therefore, it was labeled sociality. Finally, Domain 7, composed solely of the typicality factor from the CAPTION, encompasses items describing typical, regular, general, and ordinary situations. As it comprises a single dimension, it was labeled *typicality*.

### 3.3 Bass-ackwards analysis

**Figure 1:**
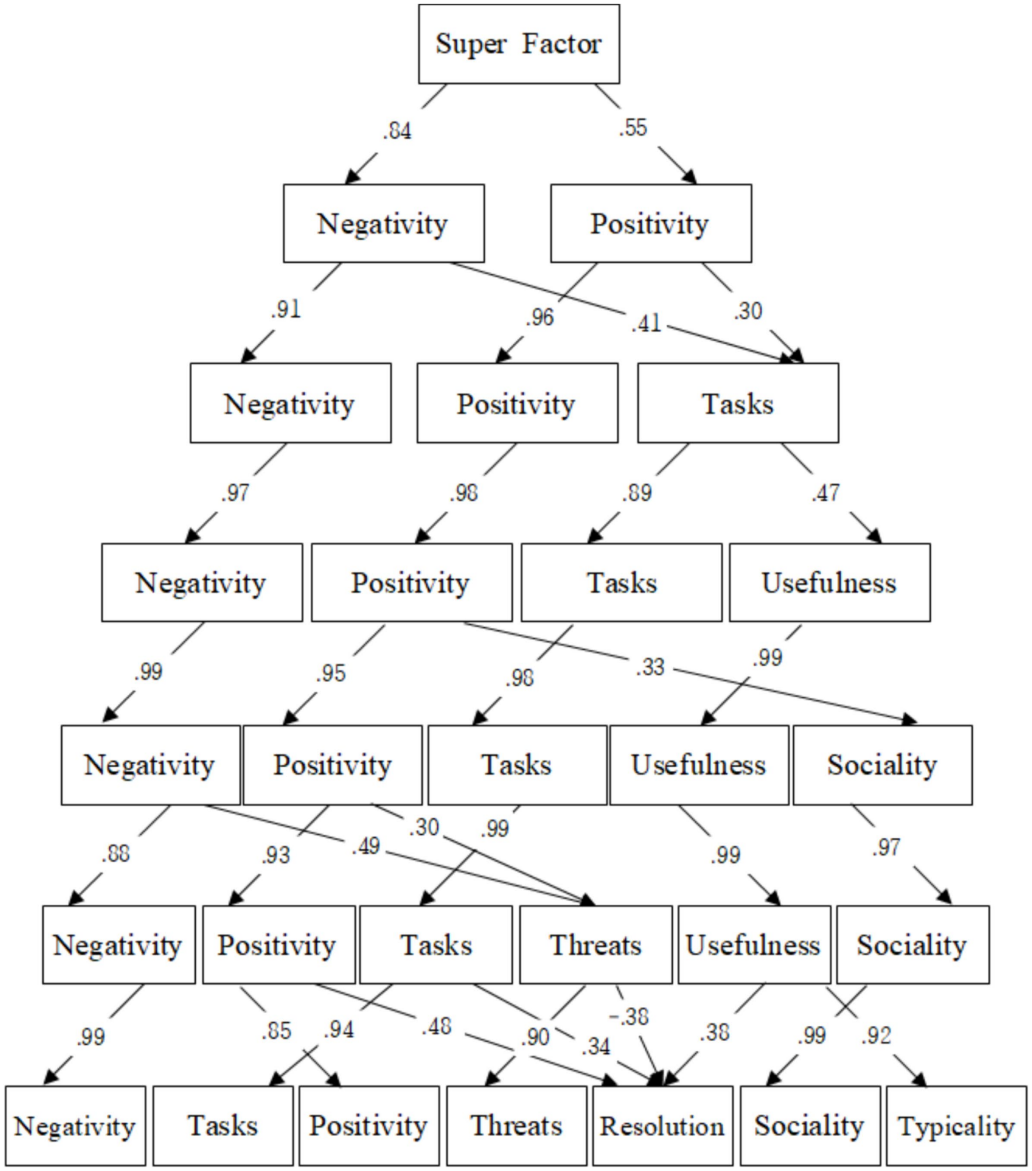
Hierarchical analysis results of the integrated situational taxonomy.

To discern the hierarchical structure of the integrated dimensions derived from the EFA of the DIAMONDS and CAPTION models targeting Korean individuals, a bass-ackwards analysis was performed (RQ2). The results of the bass-ackwards analysis are presented in Figure 1. The first step contains a common factor that encompasses all the factors. Principal component analysis of this common factor revealed that the two least correlated domains were separated in the second hierarchy. The first of these domains largely encompasses factors that induce negative emotions or situations and was labeled *negativity*. Conversely, the second domain generally involves factors that evoke positive emotions or situations, and thus was labeled *positivity*. In the third hierarchy, new factors associated with tasks and work emerged from the less-correlated factors within the negativity and positivity domains. This domain was labeled as *tasks*. The fourth hierarchy saw the tasks domain split into two: the first relating to situations requiring cognitive and academic prowess, hence labeled *tasks*, and the second composed of factors indicating beneficial and productive everyday situations, labeled *usefulness*. In the fifth hierarchy, the sociality component from the DIAMONDS model separated from the positivity domain to form an independent domain, labeled *sociality.* In the sixth hierarchy, the original negativity domain split into two: the first, encompassing core factors that describe situations inducing negative emotions, retained the *negativity* label. The second, related to threatening situations in romantic relationships, was labeled *threats*. In the seventh hierarchy, the complexity factor from the CAPTION model separated from the tasks domain and together with the importance factor from the usefulness domain, formed a new domain linked to addressing present or aimed-for situations effectively and analytically. This was labeled *resolution*. With the separation of the importance factor from the usefulness domain, only the typicality factor remained; thus, this domain was relabeled *typicality*. Ultimately, through the hierarchical analysis, we identified the domains negativity, tasks, positivity, threats, resolution, sociality, and typicality.

## 4 Discussion and conclusion

The dimensions constituting the validated DIAMONDS and CAPTION models were integrated using a domestic Korean sample. A unified situational taxonomy was derived through factor analysis and the bass-ackwards approach. This section delves into the significance of the integrated situational taxonomy derived using a Korean sample, examining in detail what it entails. Specifically, it explores the similarities and differences between the findings of this study and those from prior research conducted with Western samples (RQ3).

To integrate the DIAMONDS and CAPTION models into a single model, EFA was conducted on the factors constituting each model. Although the results identified seven domains consistent with those presented by Rauthmann and Sherman (2019), differences between this study and earlier findings were identified. For instance, based on the conceptual similarity and correlations of factors, prior studies anticipated that the integration of the DIAMONDS and CAPTION models would yield either five or six domains (Rauthmann and Sherman, 2018). However, both a previous study that attempted a factor analysis between the factors of the DIAMONDS and CAPTION models (Rauthmann and Sherman, 2019) and the current study identified seven domains from the integrated models. Although both studies extracted seven domains, discrepancies were found in the specific factors that constituted a single domain. Differences in the factors constituting the integrated domains derived from studies employing correlation analysis (Rauthmann and Sherman, 2018) and factor analysis (Rauthmann and Sherman, 2019) and from this study are presented in Table 4.

**Table 4:** Comparison of factors by domain in the integrated study of DIAMONDS and CAPTION models.

	Correlation study (Rauthmann and Sherman, 2018)	Factor analysis study (Rauthmann and Sherman, 2019)	This study
Domain 1 (negativity)	(D) negativity (C) adversity	(D) negativity (D) deception (C) adversity	(D) negativity (D) deception (C) adversity (C) negative valence
Domain 2 (tasks)	(D) duty (C) importance	(D) duty (C) importance	(D) duty (D) intellect
Domain 3 (positivity)	(D) mating (D) positivity (D) Sociality (C) positive valence (C) humor	(D) positivity (C) positive valence (C) humor	(D) positivity (C) positive valence (C) humor
Domain 4 (threats)	(D) adversity (D) deception (C) negative valence	(D) mating (D) adversity (C) negative valence	(D) mating (D) adversity
Domain 5 (resolution)	(D) intellect (C) complexity	(D) intellect (C) complexity	(C) importance (C) complexity
Domain 6 (sociality)	-	(D) sociality	(D) sociality
Domain 7 (typicality)	(C) typicality	(C) typicality	(C) typicality

(D), DIAMONDS model; (C), CAPTION model.

In Domain 1 (*negativity*), both our study and previous research consistently include the negativity dimension from the DIAMONDS model and the adversity dimension from the CAPTION model. This suggests that the core dimensions measuring negative emotions are negativity from DIAMONDS and adversity from CAPTION.

In contrast to the previous research, our study found that CAPTION’s negative valence, which was not included in the negativity domain in previous research, was included in the negativity domain. This may be attributed to the relationship between CAPTION’s adversity and negative valence. Negative valence refers to situations where specific threats are perceived from certain circumstances or entities, often resulting in negative emotions. While not all adversity situations arise from negative valence, situations characterized by negative valence are likely to be perceived as adversity situations. However, despite the similar relationship between negativity and adversity factors in the DIAMONDS model, the adversity factor in the DIAMONDS model was not included in Domain 1. This suggests that negativity and adversity in the DIAMONDS model are recognized as distinct factors. This discrepancy in results could stem from differences in the characteristics of items in the RSQ-8 and CAPTIONs-SF, as well as potential issues during the translation process. Specifically, the items in the DIAMONDS’s adversity factor describe specific situations (e.g., “Being under threat.”), whereas the negativity items focus on the negative emotions that may arise from situations (e.g., “Situation is anxiety-inducing.”). This distinction creates pronounced differences between the two factors. In contrast, both negative valence and adversity items in the CAPTION model are expressed using negative adjectives to describe negative situations. Consequently, during the translation process, fully capturing the subtle nuances of English adjectives in Korean words may have been challenging, hindering distinguishing between the characteristics of the two factors. Therefore, CAPTION’s negative valence may have been included in Domain 1.

Domain 2 (*tasks*) includes the duty and intellect dimensions from the DIAMONDS model. In contrast to our study, prior research has consistently classified both the duty dimension of the DIAMONDS model and the importance dimension of the CAPTION model in the task-related domain (Rauthmann and Sherman, 2018, 2019). In our study, the duty dimension from DIAMONDS was grouped under the same domain as intellect. This classification, which emphasizes cognitive and intellectual activities, may be attributed to the characteristics of our sample, which comprised college-educated office workers aged 25–39 years. As office jobs often involve more cognitive tasks than manual or creative labor, this specific demographic likely influenced our findings.

In Domain 3 (*positivity*), direct measures of positive emotions, such as the positivity dimension from DIAMONDS and positive valence and humor factors from CAPTION, were included. Both the correlational and factor analysis studies consistently classified these dimensions under the positivity domain, thus underscoring their centrality.

Domain 4 (*threats*) pertains to situations wherein one directly receives negative influences like criticism, condemnation, or dominance. Previous research expected this domain to comprise the adversity and deception dimensions from the DIAMONDS model and the negative valence dimension from CAPTION (Rauthmann and Sherman, 2018). However, in our study, only the adversity dimension from DIAMONDS was categorized under the threats domain. Both our study and the prior factor analysis study identified the mating dimension from DIAMONDS as a part of the threats domain. This might be because most items in the mating dimension of DIAMONDS directly inquire about sexual interactions, which are often perceived as threatening or coercive (Rauthmann et al., 2014).

Additionally, beyond these reasons, the inclusion of the mating dimension in the treats domain can be seen as reflecting characteristics of the Korean culture. The results of this study imply that romantic situations are perceived as threatening by unmarried Korean men and women in their 20s and 30s. For example, in recent Korean society, there is an ongoing trend among the youth of the 2030 age group to avoid relationships due to economic issues and self-development reasons. This age group has been increasingly referred to as the “Triple Give-up Generation” that avoids dating, marriage, and childbirth, indicating a sustained negative attitude toward these aspects among young Koreans (Korea Population, Health and Welfare Association, 2022). Furthermore, in a survey on the attitudes toward marriage and dating among the unmarried of the 2030 generation, 64% responded that considering their current situation, they feel unable to marry (K-Stat, 2021). One major reason for these responses was the economic burden, suggesting that the financial stresses of preparing for marriage, such as acquiring a home and covering wedding expenses, are perceived as threatening by this generation. Additionally, for married individuals in their 20s and 30s, economic burdens associated with conflicts with spouses, home acquisition, and increased living costs due to childcare likely contribute to the composition of the threat domain. Moreover, the increasing tendency of the Korean 2030 generation to prioritize professional achievements, personal development, and hobbies over investing time, economic, and psychological resources in relationships may be a factor in perceiving dating or marriage as threatening situations (Korea Population, Health and Welfare Association, 2022).

Domain 5 (*resolution*) relates to addressing given situations and future objectives through useful and analytical means. This domain comprises the importance and complexity dimensions of the CAPTION model. Although prior research grouped the intellect dimension from DIAMONDS model with the complexity dimension from CAPTION (Rauthmann and Sherman, 2019), our study’s differing results can be linked to our sample’s attributes. Our sample’s perception of academic and educational situations as productive and useful signifies that their generation—adept at making use of mobile technologies and diverse learning opportunities—prioritizes self-improvement.

Lastly, domains 6 (*sociality*) and 7 (*typicality*) exclusively constitute independent factors in our study and in the previous factor analysis study. While correlation studies have anticipated the sociality dimension of DIAMONDS to be classified under the positivity domain (Rauthmann and Sherman, 2018), not all positive emotions originate from interpersonal interactions. This could explain the separation of the sociality factor from the positive emotion domain. The typicality factor, which was not significantly correlated with the other dimensions from either DIAMONDS or CAPTION, is likely an independent domain.

To verify the hierarchical structures of the seven integrated domains, bass-ackwards analysis was conducted. The results of the hierarchical analysis indicated that the taxonomy of the seven domains provided the clearest and most valid interpretation compared with taxonomies with three to six domains. Before reaching the seven domains, the most meaningful and concise classification of the 15 factors with a statistically appropriate fit was the three domains categorized in the third hierarchy. These three domains were classified as negativity, positivity, and tasks, with factors in the same domain sharing conceptual similarities. In previous research employing bass-ackwards analysis, the three domains classified in the third hierarchy were negative, positive, and task-related situations (Rauthmann and Sherman, 2019). This mirrors the three domains defined in our study. Apart from the mating factor of the DIAMONDS model being classified under the positivity domain, all factor classifications in the above-mentioned study matched our findings. Thus, the taxonomy of the three domains in the third level aligns with previous research and local studies, which supports the assumption of cross-cultural applicability. In particular, both the negativity and positivity domains were related to the perception of affect that can be experienced in situations. Despite decades of debate, emotions generally consist of a correlated but independent two-factor structure: positive and negative affect. Furthermore, the Positive Affect and Negative Affect Schedule (PANAS; Watson et al., 1988), developed to measure positive and negative affect, has been validated across ages and nationalities, indicating that positive affect and negative affect are universal emotions regardless of cultural background (Park and Lee, 2016). Previous studies aiming to classify a comprehensive range of situations have revealed factors related to recognizing positive and negative affect (Moon, 2022). These previous findings indicate that while the emotions experienced in specific situations may vary depending on culture, the positive and negative affect from situations is a universal experience across cultures. Therefore, although the taxonomy of the three domains does not encapsulate specific situational characteristics, such as the seven domains, if one seeks a culturally universal, statistically clear, and concise structure of situational taxonomy, the three-domain taxonomy effectively presents an alternative to the seven domains.

Although prior research has classified the typicality factor of the CAPTION model as part of the tasks domain in the third layer, it has been consistently identified as an independent factor from the fourth layer onward; thus, it was interpreted as distinct from the three domains (Rauthmann and Sherman, 2019). However, in this study, the typicality factor was classified within the same domain as importance in the CAPTION model up to the sixth layer, and only in the seventh layer was it finally categorized as an independent domain. This suggests that the independence of the typicality factor is not as pronounced as suggested in previous research. These findings imply that the most common and repetitive situations faced by the participants in this study are those that require effective handling of given tasks and duties. This reflects that the sample population, adults in their late twenties to thirties, is in the establishment stage of Super’s career development phases, a period focused on exploring suitable job roles and securing a stable professional position (Super, 1953). Additionally, these results may reflect the Korean cultural emphasis on the importance of work activities, which places a higher value on occupational endeavors compared to Western cultures (Ryu, 2014).

This study identified seven domains that can classify a wide range of situations experienced in daily life. One suggestion is to develop an independent measurement tool capable of assessing the integrated situational taxonomy. Although this study derived the seven domains based on the common characteristics among the DIAMONDS and CAPTION model factors, the factors comprising each domain have distinct attributes. For instance, differences exist in the specificity of the situations referred to by factors (e.g., difference in the range of situations referred to by DIAMONDS’ deception and negativity), and factors that describe the situation (e.g., CAPTION’s humor) may be mixed with those that abstractly describe how the situation arises (e.g., CAPTION’s typicality). A new measurement tool composed of items that measure the core attributes of the seven domains could developed to mitigate these differences. This would allow for the utilization of the integrated situational taxonomy while alleviating the issues arising from the differences in the attributes of the factors comprising each domain.

### 4.1 Implications

This study has the following implications: One significant benefit of developing an integrated situational taxonomy is the ability to identify individual differences in situational perception. Before the development of Wave 2 situational taxonomies, clear criteria or measurement tools for individual situational perceptions did not exist. With the emergence of Wave 2 taxonomies, comparing individual or cultural differences in situational perceptions has become feasible.

Previous situational taxonomic research has primarily focused on Western cultures, with scant research across diverse cultures (Horstmann et al., 2018; Rauthmann et al., 2020). Our study is the first situational taxonomic research outside the United States and European cultural spheres. It confirms that an integrated situational taxonomy is similarly applicable in East Asian cultures.

Integrating the situational taxonomy resolved the jingle-jangle fallacy occurring between situational dimensions in different models. This fallacy arises when different concepts are given the same label (e.g., adversity in both the CAPTION and DIAMONDS models) or when similar concepts receive different names (e.g., adversity in the CAPTION and negativity in the DIAMONDS models). Through this study, the dimensions previously labeled the same despite their differing characteristics now belong to distinct domains with unique classifications. Similarly, dimensions with analogous meanings but different labels were unified under the same domain after the integrated analysis.

### 4.2 Limitations and recommendations

The limitations and recommendations of this study are as follows: First, the ultimate objective of situational taxonomy research is to describe situations in a manner that aids in explaining, understanding, and predicting human behavior. Achieving this requires a comprehensive situational taxonomy encompassing a diverse array of everyday situations. However, owing to practical constraints, this study was limited to the validation and integration of only the DIAMONDS and CAPTION models among the many developed situational taxonomies. Future research should verify whether models other than DIAMONDS and CAPTION are also applicable to the local culture, which will help in developing an integrated situational taxonomy that includes more dimensions.

Second, this study aimed to minimize the influence of participants’ demographic variables on the research results through a homogenous sample. The sample included working adults aged 25–39 years with at least a college degree. However, given that the experience of the eight situational dimensions of the DIAMONDS model may vary based on age and gender (Brown and Rauthmann, 2016), further research should verify the repeatability of these results using samples of participants with different demographic characteristics.

Finally, although the study’s main aim was the development of an integrated situational taxonomy, the ultimate goal of classifying situation characteristics was to systematically understand the influence of situations on human behavior. Once a consensus on an integrated situational taxonomy is reached, a detailed exploration of how situational characteristics interact with personality traits to influence human behavior is required. Future research can enhance our understanding of the specific interactions between situations and personalities, providing a broader comprehension of human behavior.

## Data availability statement

The datasets used and/or analysed during the current study are available from the corresponding author on reasonable request.

## Ethics statement

This study was approved by the institutional review board of Ewha Womans University (Approval No: [ewha-202110-0008-01]). The studies were conducted in accordance with the local legislation and institutional requirements. The participants provided their written informed consent to participate in this study.

## Author contributions

HM: Writing – original draft, Writing – review & editing. HA: Writing – review & editing, Supervision.
